# Hydrogen Sulfide: A Key Role in Autophagy Regulation from Plants to Mammalians

**DOI:** 10.3390/antiox11020327

**Published:** 2022-02-08

**Authors:** Angeles Aroca, Cecilia Gotor

**Affiliations:** Institute of Plant Biochemistry and Photosynthesis, University of Seville and CSIC, 41092 Seville, Spain; gotor@ibvf.csic.es

**Keywords:** autophagy, autophagy-related genes (ATG), hydrogen sulfide, persulfidation

## Abstract

Autophagy is a degradative conserved process in eukaryotes to recycle unwanted cellular protein aggregates and damaged organelles. Autophagy plays an important role under normal physiological conditions in multiple biological processes, but it is induced under cellular stress. Therefore, it needs to be tightly regulated to respond to different cellular stimuli. In this review, the regulation of autophagy by hydrogen sulfide is described in both animal and plant systems. The underlying mechanism of action of sulfide is deciphered as the persulfidation of specific targets, regulating the pro- or anti-autophagic role of sulfide with a cell survival outcome. This review aims to highlight the importance of sulfide and persulfidation in autophagy regulation comparing the knowledge available in mammals and plants.

## 1. Introduction

The term “autophagy”, (from the Greek words *auto*, meaning “self” and *phagein*, meaning “to eat”)—literally, eating one’s self—was first created by Christian de Duve over 40 years ago, who discovered lysosomes and provided clear proof of their participation in this process [1]. It is an evolutionarily conserved process of degradation and recycling in eukaryotic organisms. Two common forms of autophagy have been described in mammals and plants: micro-autophagy and macro-autophagy, while they differ in a third type of autophagy described, chaperone-mediated autophagy (in mammals) and mega-autophagy (in plants) [2,3,4,5,6]. The differences among them have been previously described in detail elsewhere [7,8], and this review will focus on macro-autophagy (hereafter, autophagy). In this latest process, the cytoplasm and/or organelles are isolated in double membrane vesicles—named autophagosomes—and then transported to the lytic organelle (vacuole in plants and yeast, and lysosome in animals) to be degraded, resulting in the turnover of cellular components. Therefore, autophagy is a fundamental cell clearance pathway that eliminates cellular components, including nucleic acids, proteins, lipids, and organelles, to promote homeostasis, differentiation, development and cell survival.

Autophagy is a unique membrane trafficking process that involves the de novo formation of a membrane, which is generally derived from the endoplasmic reticulum (ER) by generating a double membrane structure called phagophore that elongates to sequester cytoplasmic cargo and closes to form the autophagosome [6,9].

The molecular process of autophagy was mostly unknown until 1993, when Yoshinori Oshumi described a genetic screen in yeast, leading to the discovery of AuTophagy-related Genes (ATG) [10]. 41 yeast ATG genes have been described, and many of them have orthologues in other organisms such as humans and plants.

The autophagy core process in mammals is induced in response to stress by inhibiting the mammalian kinase target of rapamycin (mTOR) or activating 5’ AMP-activated protein kinase (AMPK). In mammals, different stress stimuli can trigger autophagy, such as protein misfolding, hypoxia, nutritional and energy deficiency, ER stress, redox stress, mitochondrial damage, and pathogen infection [11]. Dysregulated autophagy plays an important role in many pathological processes, including ischemia-reperfusion injury, inflammatory and infectious diseases, obesity and type 2 diabetes, cancer and neurodegenerative diseases [12,13,14].

In plants, inhibition of TOR, usually induced by starvation of nutrients such as nitrogen starvation, is the main pathway that triggers autophagy. In addition, it can also be regulated by repression of glucose signaling, activating the energy sensor Snf1-related protein kinase 1 (SnRK1), which in turn inhibits TOR and activates the ATG1 autophagy initiation complex. The function of AMPK in plant autophagy remains largely unknown, although a plant ortholog of mammalian AMPK, named KIN10, was described as a positive regulator of plant autophagy [15]. In plant cells, autophagy is triggered by different biotic and abiotic stresses such as oxidative stress, salinity, hypoxia, heat and cold, nutrient starvation, ER stress and pathogen infection. Therefore, autophagy is essential for plants during reproductive and vegetative development, senescence, starvation, immune response and it is critical to cope with environmental stress [3,16,17]. Thus, autophagy must be tightly regulated to maintain cellular homeostasis.

Hydrogen sulfide (H_2_S) is a colorless, flammable and highly toxic gas known for its rotten egg scent at low concentrations. It has always been considered a toxic pollutant that is found naturally in sewers, stagnant or well waters, compost pits, gas wells and volcanoes. However, it is also endogenously produced in cells by different enzymes.

H_2_S is produced in animals by cystathionine β synthase (CBS, EC 4.2.1.22), cystathionine-γ-lyase (CSE, EC 4.4.1.1) and 3-mercaptopyruvate sulfurtransferase (3-MST, EC 2.8.1.2); these use cysteine or 3-mercaptopyruvate as substrates. The sulfate-reducing bacterial flora in the large intestine of animals also releases H_2_S, reaching concentrations from 0.3 to 3.4 mmol L^−1^ in the colon [18,19].

In *Arabidopsis*, the plant species where the H_2_S signaling has been deeply studied, H_2_S is produced from cysteine by the action of L-cysteine desulfhydrases (DES1, EC 4.4.1.2; and L-CDES, EC 4.4.1.1), D-cysteine desulfhydrases (D-CDES, EC 4.4.1.15), cyanoalanine synthase (CAS, EC 4.4.1.9), cysteine synthase (CS, EC 4.2.99.8), NifS-like proteins and in the photosynthetic sulfate assimilation pathway by sulfite reductase (SiR, EC 1.8.7.1) [20].

Over the last decade, both in animal and plant systems, H_2_S has been highlighted as a biological signaling molecule—namely, gasotransmitter—as important as other signal molecules such as nitric oxide (NO), carbon monoxide (CO) or hydrogen peroxide (H_2_O_2_) [21,22,23].

H_2_S is already considered a physiological mediator involved in many physiological and pathological processes in animals and plants. Its regulatory function in mammals includes processes such as reducing inflammation [24], synaptic transmission [25], apoptosis [26], vascular tone [27], ischemia-reperfusion injury [28] and promoting ulcer healing [29] and protects cells from oxidative stress [30]. In plants, H_2_S regulates a wide range of physiological processes, from seed germination to fruit maturation and the first description of its influence on vegetative development and disease resistance of plants dates from the late 1960s [31,32]. Today, the protective effects of H_2_S against different stresses are widely known, such as drought [33], osmotic and saline stresses [34], heat [35], oxidative stress [36] and metal stresses [37]. In addition, H_2_S regulates photosynthesis [38], stomatal closure/aperture [39,40] and autophagy [41,42,43,44,45,46,47].

## 2. Hydrogen Sulfide as a Regulator of Autophagy

### 2.1. The Anti-Autophagic Role of Sulfide in Plants

Over the last 10 years, there have been many studies on the effects of H_2_S on autophagy in eukaryotic cells, but its mechanism has not been completely deciphered.

**Figure 1:**
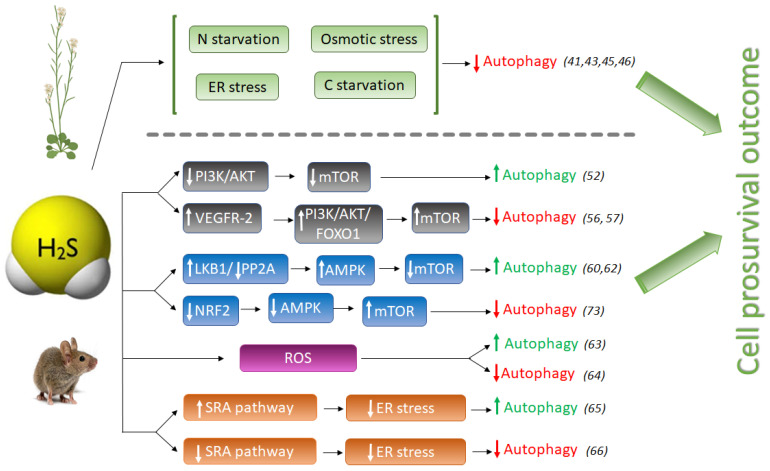
Schematic comparison of pro-autophagic and anti-autophagic effect of H_2_S signaling in animal and plant systems. H_2_S, hydrogen sulfide; LKB1, liver kinase B1; PP2A, Protein phosphatase 2; AMPK, adenosine monophosphate-activated protein kinase; mTOR, mammalian target of rapamycin; NRF2, Nuclear factor E2-related factor 2; Akt (PKB), protein kinase B; PI3K, phosphatidylinositol 3-kinase; FOXO1, Forkhead Box O1; VEGFR-2, Vascular endothelial growth factor receptor 2; SRA, Scavenger receptor A. Numbers between brackets refer to references cited.

In plants, the role of H_2_S in autophagy has been described as a protective effect towards a prosurvival outcome. By now, H_2_S has been revealed as a negative regulator of autophagy induced by nutrient deficiency, carbon and nitrogen deprivation [41,43] and osmotic [46] and ER stress [45] (Figure 1). It was shown that only sulfide donor molecules, and no other compounds containing inorganic sulfur, are responsible for the inhibition of autophagy under nitrogen starvation in Arabidopsis roots [43]. Besides, sulfide signaling was dose-dependent, with an optimal NaHS (commonly used as sulfide generating molecule) concentration of 100–200 µM, with devastating effects at higher concentrations, inducing autophagy, probably due to its toxicity.

The induction of autophagy by oxidative stress, especially during nutrient deprivation, and the ability of H_2_S to activate the antioxidant response of plant cells, are well known. However, it was nicely shown that the negative regulation of autophagy through sulfide signaling was not dependent on its antioxidant activity, showing that hydrogen sulfide does not behave as an H_2_O_2_ or superoxide scavenger [43]. Furthermore, treatments with identical concentrations of antioxidant molecules such as glutathione and ascorbate were unable to produce the negative effect that sulfide treatment had on autophagy regulation; only NaHS treatment significantly inhibited autophagy [43].

Hydrogen sulfide has also been revealed to play a key role in autophagy during ER stress. Aggregation of misfolded proteins in the endoplasmic reticulum disrupts ER function, producing ER stress [48], which interferes with normal physiological functions of the cell. ER stress occurs when an increase of misfolded proteins accumulate in the ER which may be activated by different adverse environmental conditions such as cold or heat and pathogen infections in plants [49], or by several chemical and physiological situations such as glucose deprivation, hypoxia or genome instability in animals [50].

In a recent study, the effect of sulfide was also demonstrated to be independent from the antioxidant activity under ER stress, comparing the results observed using similar concentrations of sulfide, glutathione and ascorbate. Their results showed that when ER stress was induced with tunicamycin, no significant decrease in autophagosomes was detected upon well-established antioxidant compounds. By contrast, sulfide provoked a severe decrease of autophagosomes, indicating that the negative effect of sulfide is independent of redox conditions [45].

The anti-autophagic role of sulfide in *Arabidopsis* was also demonstrated under induced autophagy by carbon starvation, where Cys-generated sulfide in the cytosol was shown to regulate negatively autophagy and to modulate the transcriptional profile of Arabidopsis [41]. DES1, the L-Cys desulfhydrase protein located in the cytosol, catalyzes the desulfuration of L-Cys to sulfide plus ammonia and pyruvate. Consequently, the null mutant *des1-1* plants contain 30% less endogenous sulfide in leaves than WT plants. Mutant *des1-1* plants were shown to have induced autophagy under physiological conditions, and exogenous treatment with NaHS negatively regulated autophagy in this mutant background [41]. Moreover, sulfide was able to suppress autophagy induction caused by carbon starvation even in wild-type plants, whereas exogenous ammonia, also a product of DES1 activity, had no effect on carbon-induced autophagy. Therefore, it was concluded that sulfide exerts a general effect on autophagy unrelated to nutrition limitation stress.

In a different study sought to decipher the mechanism of action by which NaHS regulates autophagy, it was shown that abscisic acid (ABA) treatment induced the autophagic flux and that this induction was also repressed by NaHS [46]. One of the first plant responses to adverse environmental conditions is the increase of intracellular ABA content in order to activate downstream ABA-signaling pathway so as to help plants cope with the stress. In this situation, when plants successfully have overcome the adverse conditions and induced autophagy is not more required, NaHS repression prevents the over-activation of autophagy allowing to return back to levels in favorable growth conditions [44]. Therefore, in all studies reported up to now in plant systems, sulfide has an anti-autophagic role (Figure 1).

### 2.2. The Pro- or Anti-Autophagic Role of Sulfide in Mammals

However, the pro- or anti-autophagic role of H_2_S in mammals has not always been completely clear, and several publications have shown that autophagy and H_2_S could be a double-edged sword in cancer studies depending on the experimental settings. Hydrogen sulfide induces autophagy of hepatocellular carcinoma cells (HCC) by inhibiting the phosphatidylinositol 3-kinase (PI3K)/protein kinase B (AKT)/ mTOR (PI3K/Akt/mTOR) signaling pathway [51]. PI3Ks are a family of lipid kinases, which phosphorylate phosphoinositides that entail AKT recruitment to the cell membrane. AKT is an evolutionarily highly conserved serine/threonine protein kinase, considered one of the key downstream proteins of PI3K. mTOR is a conserved serine/threonine protein kinase and it is the catalytic core of two complexes: mTORC1 and mTORC2. Activation of the PI3K/Akt pathway further phosphorylates downstream regulators such as mTOR and the transcription factor Forkhead box O-1 (FoxO-1), upregulating the activity of mTOR complex 1 (mTORC1) that drives autophagy inhibition [52]. The PI3K/AKT signaling pathway is one of the upstream pathways that regulate mTOR. Suppression or dysfunction of PI3K can greatly block the downstream signaling pathways AKT and mTOR, and therefore the induction of autophagy (Figure 1).

NaHS treatment significantly inhibited the expression of phospho-PI3K, phospho-Akt, and mTOR proteins in HCC cells, mimicking the effect of rapamycin [51], and therefore activating autophagy. However, NaHS did not affect basal-level Akt phosphorylation in heart disease during ischemia, but further doubled myocardial Akt phosphorylation during reperfusion [53]. Zhou Y. et al., also found that NaHS enhances mTOR phosphorylation in both ischemic and reperfused hearts [53]. In another study, pretreatment of MC3T3-E1 osteoblasts with SDSS [a H_2_S donor derived from β-(3,4-dihydroxyphenyl)lactic acid] stimulated Akt phosphorylation in a concentration-dependent manner [54]. H_2_S also activates vascular endothelial growth factor 2 (VEGFR-2), which, in turn, activates the PI3K/AKT/FOXO-1 signaling pathway, with the opposite result of inhibition of autophagy [55,56] (Figure 1).

Thus, the role of NaHS activating the phosphorylation or dephosphorylation of the PI3K/AKT signaling pathway and the outcome of autophagy regulation has not been clearly deciphered in mammals. The different cell types, as well as the sulfide concentrations used in the experiments, were likely the consequence of different conclusions.

The adenosine monophosphate-activated protein kinase (AMPK) is involved in the regulation of metabolic energy balance, and several studies have implicated the AMPK/mTOR pathway in the regulation of autophagy. Numerous publications described the role of H_2_S in activating autophagy through the AMPK/mTOR pathway, making this signaling a promising target for several diseases [18,57,58].

Another pro-autophagic effect of H_2_S has been described in the regulation of the liver kinase B1 (LKB1)-AMPK signaling pathway [59]. LKB1 forms a heterotrimeric complex with the pseudokinase Ste20-related adaptor (STRAD) and the scaffolding mouse protein 25 (MO25), and this LKB1-STRAD-MO25 complex activates AMPK by phosphorylation [60]. Kundu et al. described that NaHS treatment in hyperglycemic cells increased LKB1/STRAD/MO25 complex assembly and therefore, AMPK phosphorylation, promoting autophagy [59] (Figure 1). However, a later study demonstrated that H_2_S regulated AMPK phosphorylation through inhibition of protein phosphatase 2A (PP2A), and not through the LKB1/STRAD/MO25 complex [61], but sulfide still had a pro-autophagic role.

The protective effect of sulfide in several illnesses has been linked to its role promoting autophagy which may decrease ROS production. In a recent study in endothelial progenitor cells (EPCs), exogenous H_2_S ameliorated the high glucose (HG)-induced injury by promoting autophagic flux and decreasing ROS production, demonstrating the protecting role of sulfide under this dysfunction [62]. Their findings demonstrated that the phosphorylation of the endothelial nitric oxide synthase (eNOS) at Thr495 determines whether this enzyme produces either NO or superoxide, and sulfide reduced the phosphorylation level of this enzyme, decreasing NO and ROS production [62].

But on the other hand, it is well known that oxidative stress may induce autophagy to protect cells from apoptosis. The effect of sulfide ameliorating oxidative stress has also been described in mice where it was demonstrated that GYY4137, a sulfide donor, attenuated the severity of lung injury by alleviating septicemia-induced ferroptosis and inhibiting the activation of autophagy in sepsis-induced acute lung injury [63]. Therefore, sulfide may play an anti-autophagic role by alleviating oxidative stress (Figure 1).

Prolonged ER stress has been associated with a wide range of diseases, including neurodegeneration, cancer, atherosclerosis, type 2 diabetes and liver disease. Autophagy is activated to remove dysfunctional proteins during ER stress. Several studies have connected the role of sulfide enhancing autophagy in reducing ER stress in mammals. In peritoneal macrophages of rats, hydrogen sulfide induces autophagy by suppressing the class A scavenger receptor (SRA) pathway (Figure 1). This cell response reduces ER-stress by inducing autophagy and protects against ischemia/reperfusion injury, maintaining liver function [64]. In other studies, NaHS treatment blocked ER stress and ER stress-associated autophagy [65].

During ER stress, H_2_S has been reported to inhibit protein tyrosine phosphatase (PTP1B) [66]. PTP1B dephosphorylates PKR-like endoplasmic reticulum kinase (PERK), an ER stress sensor that autophosphorylates and induces the phosphorylation of eukaryotic initiation factor 2 alpha (eIF2α), which is necessary to mediate the induction of autophagy [67]. Therefore, it was concluded that exogenous H_2_S, or induction of its endogenous synthesis, suppress the activation of PERK/eIF2α/ATF4 pathway and its subsequent effects on ER stress, which are an increased eIF2α phosphorylation [68], and the induction of autophagy. In addition, H_2_S suppresses the expression of PKR-like endoplasmic reticulum kinase (PERK) [69], which induces autophagy.

Additionally, H_2_S induces the activity of the transient receptor potential channel (TPRV4) and KATP channels, mediating angiogenesis and inducing vasodilation [70,71]. Through activation of TPRV4, H_2_S also activates the AMPK/mTOR pathway, by this means reducing autophagy [72].

Hydrogen sulfide also exerts a cytoprotective role by upregulating cellular antioxidants by suppressing nuclear factor erythroid-2 related factor 2 (NRF2) [73]. NRF2 is a family of nuclear basic leucine zipper transcription factors that regulate the gene expression of a number of antioxidant enzymes. However, Nrf2 can also sense ROS and RNS in stressed cells, triggering the activation of AMPK, which suppresses mTOR and therefore induces autophagy [74]. Thus, NaHS could also inhibit excessive autophagy of vascular endothelial cells by the Nrf2/AMPK signaling pathway [72].

We can draw the conclusion that in mammals H_2_S could play opposite effects, enhancing or decreasing autophagy induction, which may be attributed to the sulfide concentration, reaction time, cell types and/or differences among the diseases studied. The administration of exogenous H_2_S in mammalian systems has also typically been performed at micromolar concentrations as in plants [75]. Higher doses of H_2_S exposed in some publications lead to contradictory data.

However, in all cases, the final outcome of the role of H_2_S is cell survival, which likewise has been described in plant systems. When stress is mild, in mammals H_2_S often activates autophagy to protect cells, usually by reducing stress conditions, but with the progression of the disease, H_2_S can act as a regulator inhibiting autophagy to avoid excess stress-induced autophagy and cell death.

## 3. Persulfidation as the Molecular Mechanism of Sulfide for Autophagy Regulation

During the past decade, the research of H_2_S as a signaling molecule has been focused on the effect of sulfide donors on different diseases and physiological pathways, until in 2009 when Snyder’s group described persulfidation or S-sulfhydration as the mechanism of H_2_S signaling [76]. Since then, numerous targets have been identified to undergo persulfidation, and it has become recognized as the main mechanism by which H_2_S controls several cellular functions. Persulfidation is a posttranslational modification of cysteine residues, where a thiol group (RSH) is transformed into a persulfide group (RSSH). Modified cysteines show greater reactivity than their thiol counterparts [23]. It has been proven that this new posttranslational oxidative modification can affect the subcellular localization of the modified protein [77], its activity [76,78] and stability, and it has been proposed to be a cellular mechanism to cope with oxidative stress [79,80]. Numerous studies have demonstrated that persulfidation is a widespread modification in animal and plant cells [76,81] involved in a huge range of biological processes [82], which explains the great interest of the scientific community in understanding this cell mechanism.

### 3.1. Regulation of Autophagy by Persulfidation in Plants

The role of persulfidation as the molecular mechanism of sulfide for autophagy regulation was first proposed in plants by Gotor’s group [83] and then several autophagy-related core proteins were demonstrated to be targets for persulfidation [82,84]. Recently, the autophagy-related (ATG) proteins, ATG18a, ATG3, ATG5 and ATG7 were published to be targets for persulfidation identified by a quantitative proteomic study in *Arabidopsis* leaves [82]. Nevertheless, the role of persulfidation in those proteins was not deciphered.

In a very recent quantitative proteomic approach in Arabidopsis under nitrogen deprivation, more than 5200 proteins were identified as targets for persulfidation. In this work, authors extended the number of persulfidated proteins involved in autophagy. They found 17 proteins that play an essential role in core autophagy machinery were persulfidated; including ATG2, 3, 4, 5, 7, 11, 13, the serine/threonine kinase TARGET OF RAPAMYCIN (TOR), its effectors proteins REGULATORY-ASSOCIATED PROTEIN OF TOR 1 (RAPTOR 1) and LETHAL WITH SEC THIRTEEN PROTEIN 8 (LST8), five subunits of PP2A, the regulatory subunit of PP2A (TAP46) and the serine/threonine-protein kinase VPS15 [84]. In addition, this study also revealed that other 58 proteins related to endocytosis and the formation of the phagophore were persulfidated, including several transporters and vacuolar sorting proteins.

**Figure 2:**
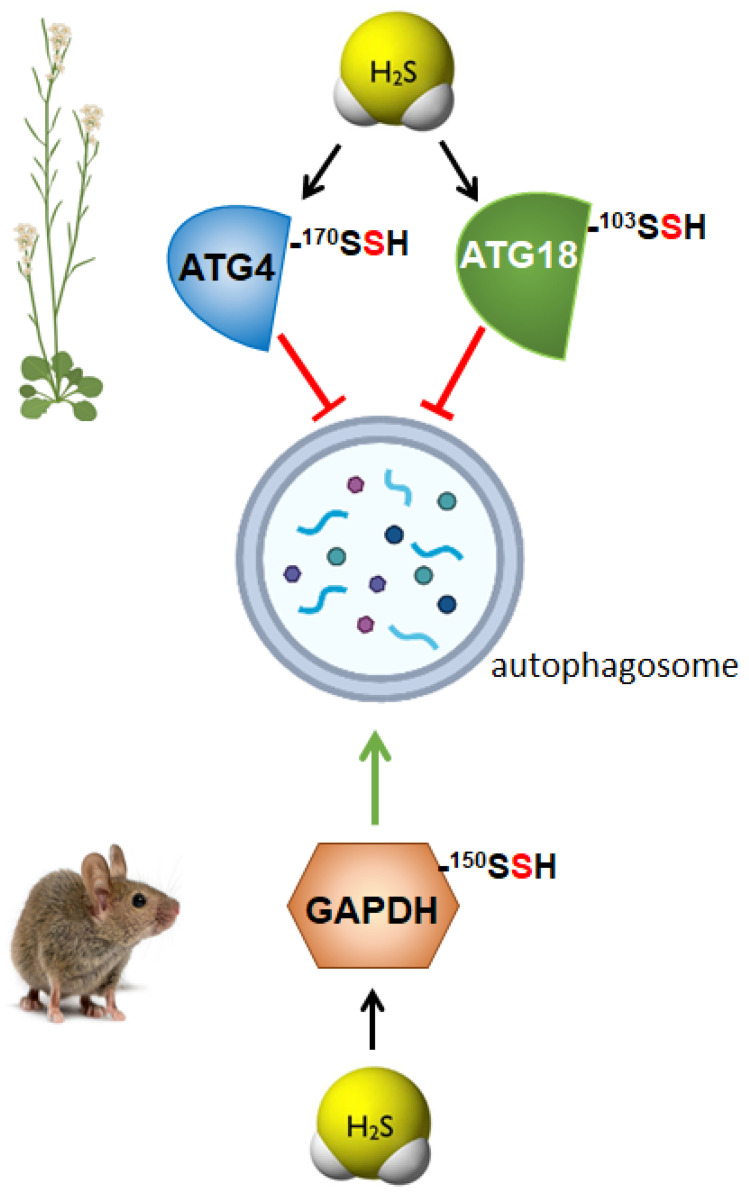
Persulfidation of specific proteins differently regulates autophagy in plants and mammals. ATG4, autophagy-related gene 4; ATG18, autophagy-related gene 18; GAPDH, glyceraldehyde-3-phosphate dehydrogenase.

The role of persulfidation as the underlying mechanism regulating autophagy through sulfide was demonstrated in the ATG4 protease, which was specifically modified by persulfidation of Cys170 residue, negatively regulating [46] (Figure 2). These authors established that persulfidation of ATG4 upon sulfide treatment inhibited its protease activity, disabling the progress of autophagy. They also revealed that an increase in the intracellular level of the plant hormone abscisic acid (ABA) triggered a decrease in the persulfidated ATG4 level; consequently, its protease activity was enhanced, activating the processing of ATG8, which was further lipidated, and, as a result, autophagy was induced.

In plants, the role of H_2_S in the regulation of autophagy has been studied under stress conditions, particularly under nutrient limitation, demonstrating the negative regulation of bulk autophagy by sulfide through persulfidation of specific targets [41,46,85]. However, a recent research was published describing the role of persulfidation of ATG18a regulating autophagy under ER-stress [45], and, therefore, deciphering a new level of regulation of selective autophagy through the persulfidation of ATG18a [45] (Figure 2). ATG18a binds to phosphoinositides [86] and forms a complex with ATG2, which is involved in autophagosome biogenesis during phagophore expansion [87]. In this research, it was demonstrated that sulfide regulates ATG18a phospholipid-binding activity by reversible persulfidation at the specific residue Cys103, which reversibly activates ATG18a binding capacity to specific phospholipids. Authors proved that the mutation of Cys103 in ATG18a decreased its binding capacity to membranes and its localization time within the phagophore was shorter. In this way, the reversible persulfidation of ATG18a affects its binding to membranes, which potentially delays its release from the autophagosome, inhibiting autophagosome progression and maturation [45]. This regulation of autophagy through the persulfidation of ATG18a probably allows the plant a correct physiological response upon stress, with the final outcome of plant survival.

### 3.2. Regulation of Autophagy by Persulfidation in Animals

The molecular mechanism by which H_2_S regulates autophagy in mammals has been recently established thought the study of the persulfidation of glycolytic glyceraldehyde-3-phosphate dehydrogenase (GAPDH) [88] (Figure 2). This modification of GAPDH triggers its translocation to the nucleus, which is critical to induce autophagy via deacetylation of the autophagic core protein LC3B, and the consequential autophagosome formation. Authors demonstrated that nuclear GAPDH interacts with the cell cycle activator and apoptosis regulator 2 (CCAR2/DBC1), avoiding the interaction of CCAR2 with deacetylase SIRT1, and therefore avoiding the inactivation of SIRT1. Then, activated SIRT1 deacetylates MAP1LC3B/LC3B (microtubule-associated protein 1 light chain 3 beta) inducing its translocation into the cytoplasm and activating autophagy [88]. Persulfidation of GAPDH at the same residue, Cys150 was previously described in mammals resulting in an increase of its enzymatic activity [76], although in later studies, polysulfide treatment of GAPDH showed opposite effects decreasing its enzymatic activity [89]. In plants, persulfidation of the cytosolic isoform GAPDH (GapC) was also previously described [78] and demonstrated its nuclear translocation upon the modification of the protein [77]. However, in none of these studies, the relationship between persulfidation of GAPDH and autophagy regulation was analyzed. A similar situation comes about the regulation of protein tyrosine phosphatase (PTP1B), which was described to be persulfidated at Cys215, inhibiting its enzymatic activity. This inhibition resulted in the activation of PERK alleviating ER stress [66], but authors did not relate this regulation to autophagy. Nevertheless, later was demonstrated that activation of PERK by sulfide treatments, increased eIF2 phosphorylation and induced autophagy [68], probably due to the persulfidation of PTP1B.

## 4. Conclusions

The aim of this review is to provide insights into the role of H_2_S regulating autophagy, contrasting the available knowledge in plant and animal systems. In plants, it has been widely accepted that the beneficial effect of sulfide mitigating different stresses, as well as all the studies published, point toward an anti-autophagic role of sulfide by repressing autophagy. Accumulating experimental evidence in mammalians demonstrates the cytoprotective effect of sulfide in a wide range of physiologic and pathologic conditions and its role through regulating autophagy. However, it needs further clarification as to if sulfide exerts a pro- or anti-autophagic role in mammalians.

It is worth pointing out that the mechanisms by which hydrogen sulfide regulates autophagy are through the persulfidation of specific targets, what seems to be evolutive conserved among species.

Therefore, from all the above, we can conclude that these findings suggest that either in mammals and in plant systems, the regulation of autophagy by persulfidation of specific targets seems to be the H_2_S signaling mechanism in autophagy, and, regardless, because sulfide exerts a pro-autophagic or anti-autophagic role, its beneficial effect points toward cell survival.
